# In Vitro and In Vivo Anti-*Candida* spp. Activity of Plant-Derived Products

**DOI:** 10.3390/plants8110494

**Published:** 2019-11-11

**Authors:** Reginaldo dos Santos Pedroso, Brenda Lorena Balbino, Géssica Andrade, Maria Cecilia Pereira Sacardo Dias, Tavane Aparecida Alvarenga, Rita Cássia Nascimento Pedroso, Letícia Pereira Pimenta, Rodrigo Lucarini, Patrícia Mendonça Pauletti, Ana Helena Januário, Marco Túlio Menezes Carvalho, Mayker Lazaro Dantas Miranda, Regina Helena Pires

**Affiliations:** 1 Universidade de Franca, Franca 14404-600, SP, Brazil; rpedroso@ufu.br (R.d.S.P.); brendalorenabalbino@hotmail.com (B.L.B.); gessicaandrade16123@gmail.com (G.A.); ceciliasacardo@outlook.com (M.C.P.S.D.); tavanealvarenga@gmail.com (T.A.A.); ritinha-pedroso@hotmail.com (R.C.N.P.); leticia_pimenta94@hotmail.com (L.P.P.); rodrigolucarini@hotmail.com (R.L.); patricia.pauletti@unifran.edu.br (P.M.P.); ana.januario@unifran.edu.br (A.H.J.); 2 Escola Técnica de Saúde, Universidade Federal de Uberlândia, Uberlândia 38400-902, MG, Brazil; 3 Programa de Pós-graduação em Ciências da Saúde, Universidade Federal de Uberlândia, Uberlândia 38400-902, MG, Brazil; 4 Universidade Estadual de Minas Gerais, Passos 37902-407, MG, Brazil; marcotulioibc@outlook.com; 5 Instituto Federal do Triângulo Mineiro, Campus Uberlândia Centro, Uberlândia 38.064-300, MG, Brazil; maykermiranda@iftm.edu.br

**Keywords:** plant-derived products, *Candida*, *C. elegans*, anticandidal agents

## Abstract

Candidiasis therapy, especially for candidiasis caused by *Candida* non-*albicans* species, is limited by the relatively reduced number of antifungal drugs and the emergence of antifungal tolerance. This study evaluates the anticandidal activity of 41 plant-derived products against *Candida* species, in both planktonic and biofilm cells. This study also evaluates the toxicity and the therapeutic action of the most active compounds by using the *Caenorhabditis elegans–Candida* model. The planktonic cells were cultured with various concentrations of the tested agents. The *Cupressus sempervirens*, *Citrus limon*, and *Litsea cubeba* essential oils as well as gallic acid were the most active anticandidal compounds. *Candida* cell re-growth after treatment with these agents for 48 h demonstrated that the *L. cubeba* essential oil and gallic acid displayed fungistatic activity, whereas the *C. limon* and *C. sempervirens* essential oils exhibited fungicidal activity. The *C. sempervirens* essential oil was not toxic and increased the survival of *C. elegans* worms infected with *C. glabrata* or *C. orthopsilosis*. All the plant-derived products assayed at 250 µg/mL affected *C. krusei* biofilms. The tested plant-derived products proved to be potential therapeutic agents against *Candida*, especially *Candida* non-*albicans* species, and should be considered when developing new anticandidal agents.

## 1. Introduction

Infections caused by yeasts belonging to the genus *Candida* affect especially immunocompromised individuals, children, elderly, individuals hospitalized in Intensive Care Units (ICU), and users of invasive devices [1]. Vulvovaginitis caused by *Candida* and *Candida*-associated stomatitis also represent important infections in the field of Public Health [2,3].

Factors such as the use of immunosuppressive drugs, broad-spectrum antibiotics, and antifungal agents for prophylaxis have increased the number of patients that are susceptible to opportunistic diseases, including candidiasis, particularly candidiasis caused by non-*albicans Candida* (NAC) species such as *C. glabrata*, *C. krusei*, *C. parapsilosis*, *C. tropicalis*, and, more recently, *C. auris* [4].

Depending on the microenvironment’s nutritional content, micro-organisms, including *Candida,* can grow in the planktonic or the biofilm form. The biofilm is represented by aggregated, organized, and functional micro-organisms embedded in an exopolymeric matrix, which allows irreversible adhesion to biotic or abiotic surfaces [5]. Microbial biofilms are the main cause of hospital infections and the source of many recurrent and persistent diseases [4,5]. Furthermore, NAC species leading to infections, including species that may be resistant to more than one class of antifungal agents, have contributed to increasing the intrinsic or acquired resistance of *Candida* isolates to antifungal drugs [4,5].

The search for alternatives for the primary or complementary therapy of infections caused by *Candida* has been constant. In this context, plant-derived products allow the discovery of new agents with potential application in the clinical setting and in the development of drugs for systemic and/or topical use [6].

Plants have several secondary metabolites that display antimicrobial activity. For instance, plant essential oils (EOs) consist of various naturally associated compounds among which terpenes (monoterpenes and sesquiterpenes), aromatic compounds (aldehyde, alcohol, phenol, and methoxy derivative), and terpenoids (isoprenoids) predominate [7]. In the case of yeast-like fungi, terpenes have been reported to inhibit 3-hydroxy-3-methylglutaryl coenzyme A (HMG CoA) reductase, cell growth and signaling modulators, apoptosis initiators, and cell cycle arrest inducers [8].

In addition, essential oils may be rich in phenolic compounds, which present antioxidant properties due to their ability to act as hydrogen donors, reducing agents, singlet oxygen depleters, and metal chelators [7,9,10]. Thus, the activity of essential oils is related to the composition, functional groups, and synergistic interactions between the components [9], not to mention that the harvesting period also determines the concentrations of the main components in the plant oil [10].

Approximately half of the drugs that were approved for use between 1940 and 2006 were derived from natural products [11], which exhibited antimicrobial, anticancer, antidiabetic, and antidepressant actions, among others [10]. This highlights that plants are a potential source of bioactive molecules. However, there is limited knowledge about the activity of plant-derived products against NAC species, mainly against *C. glabrata* and *C. krusei,* which are less susceptible or resistant to fluconazole, the most widespread antifungal for prophylactic and/or therapeutic use [4].

The development of novel antifungal drugs requires both safety and toxicity assessment. Currently, in vivo studies have increasingly employed the so-called alternative models, which replace animals such as mice and rats with other animals, such as the *Danio rerio* fish, the *Galeria mellonella* larvae, and the *Caenorhabditis elegans* nematode. These alternative models are suitable to investigate acute or systemic toxicity, pharmacokinetics, and infections. *C. elegans* offers advantages that include its easy handling in the laboratory, reasonable cost, and known genome, which enables toxic compounds to be screened and their antifungal activity against *Candida* yeasts to be evaluated [12].

In this study, the antifungal action of plant-derived products against *Candida* species growing both as planktonic and biofilm cells has been investigated. In addition, the toxicity and the effectiveness of the most active compounds have been studied by using a *C. elegans–Candida* infection model.

## 2. Results

Forty-one plant-derived products were tested against six *Candida* species (Table 1). Among the tested essential oils (EOs, 30 samples), the *Cupressus sempervirens* (cypress) EO presented the best result; it acted against all the *Candida* species. The minimum inhibitory concentration (MIC) values were 250, 250, 62.5, 31.25, 62.5, and 31.25 μg/mL against *C. albicans*, *C. tropicalis*, *C. krusei*, *C. glabrata*, *C. parapsilosis*, and *C. orthopsilosis*, respectively (Table 1).

The *Citrus limon* EO provided MIC values of 250 μg/L against *C. tropicalis* and *C. glabrata* (Table 1). The *Litsea cubeba* EO yielded MIC values of 62.5, 250, and 250 μg/mL against *C. krusei, C. glabrata*, and *C. orthopsilosis*, respectively (Table 1). Finally, the *Citrus reticulata* EO afforded MIC values of 250 μg/mL against both *C. krusei* and *C. orthopsilosis* (Table 1). The first three products were tested against *Candida* in the following assays.

According to the adopted criteria for antimicrobial activity, none of the five plant extracts tested here (Table 2) were active against the evaluated *Candida* strains, their MIC values were higher than 2000 μg/mL.

Among the seven plant-derived compounds tested herein (Table 3), gallic acid showed the greatest activity; its MIC values were 125, 31.25, 250, and 250 μg/mL against *C. krusei*, *C. glabrata*, *C. parapsilosis*, and *C. orthopsilosis*, respectively, so this acid was selected for further studies. Against the *C. parapsilosis* ATCC 22019 and the *C. krusei* ATCC 6258 reference strains, amphotericin B (AMB) gave MIC values of 0.25 and 1.00 μg/mL, respectively.

The EOs were extracted from *Cupressus sempervirens* and *Citrus limon* leaves and *Litsea cubeba* fruits in 0.65%, 1.5%, and 1.0% yield, respectively. GC-MS and GC-FID analyses helped to identify 13, 9, and 11 chemical constituents in the EOs extracted from *Cupressus sempervirens* (total 99.1%) and *Citrus limon* (total 98.1%) leaves and *Litsea cubeba* fruits (total of 96.2%), respectively. Sabinene, terpinen-4-ol, citral, limonene, neral, and geraniol were the major compounds in these EOs. Table 4 lists all the identified compounds, retention indexes, and relative area percentages (% RA).

**Figure 1:**
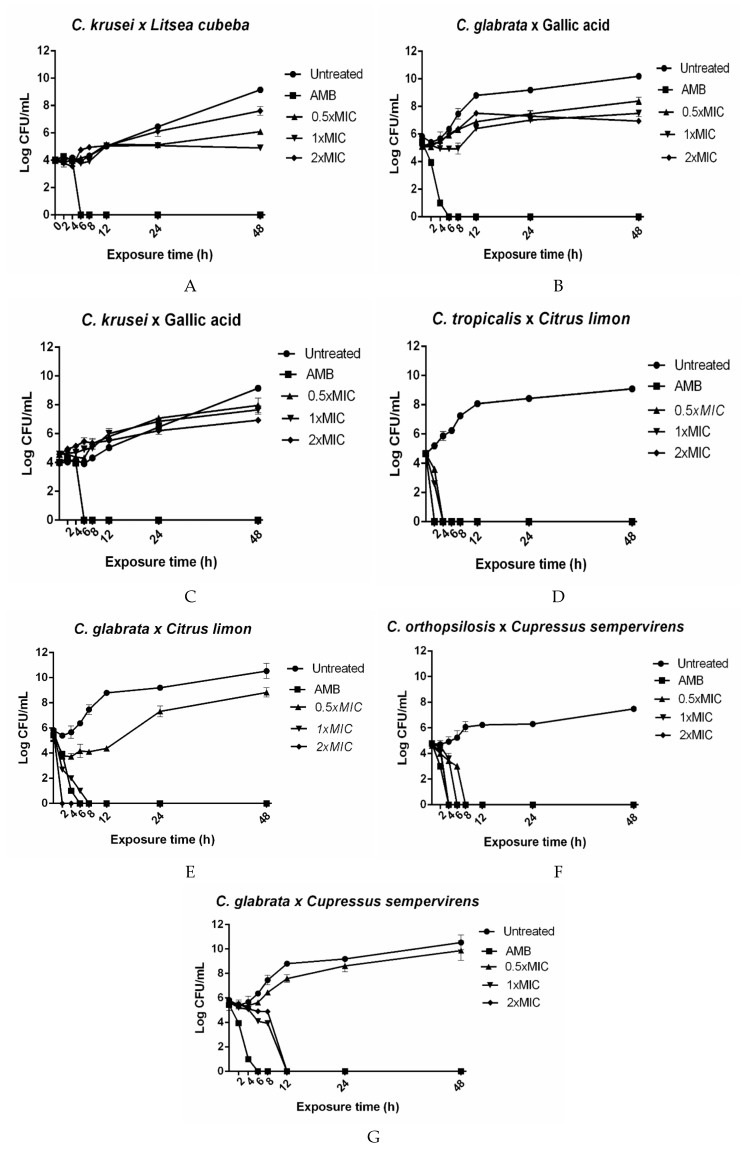
Kill assays for plant-derived products against *Candida* species. The concentrations 0.5 × MIC, 1 × MIC, and 2 × MIC correspond to: (**A**) *Litsea cubeba* × *C. krusei*: 31.25, 62.5, and 125 µg/mL; (**B**) Gallic acid × *C.glabrata* 31.25, 62.5, and 125 µg/mL; (**C**) Gallic acid × *C. krusei*: 62.5, 125, and 250 µg/mL; (**D**) *Citrus limon* × *C. tropicalis*: 125, 250, and 500 µg/mL; (**E**) *Citrus limon* × *C. glabrata*: 125, 250, and 500 µg/mL; (**F**) *Cupressus sempervirens* × *C. orthopsilosis:* 15.62, 31.25, and 62.5 µg/mL; (**G**) *Cupressus sempervirens* × *C. glabrata*: 15.62, 31.25, and 62.5 µg/mL. AMB: 4 µg/mL amphotericin B and Untreated: *Candida* species’ growth without plant-derived products. The results are expressed as the mean colony-forming units (CFU)/mL ± standard deviation from three independent experiments.

The *Litsea cubeba* EO was tested against *C. krusei*, and gallic acid was assayed against *C. glabrata* and *C. krusei*. The tested agents were fungistatic at all the concentrations (Figure 1A–C). The *Citrus limon* EO at 0.5 × MIC (125 μg/mL) or 1 × MIC (250 μg/mL) exerted fungicidal action against *C. tropicalis* (Figure 1D) after 4 h. The *Citrus limon* EO at 2 × MIC (500 μg/mL) had a fungicidal effect on *C. tropicalis* (Figure 1D) and *C. glabrata* (Figure 1E) after 2 h. A fungicidal effect emerged after exposure of *C. orthopsilosis* (Figure 1F) to the *C. sempervirens* EO at 0.5 × MIC (15.6 μg/mL), 1 × MIC (31.25 μg/mL), and 2 × MIC (62.5 μg/mL) for 8, 6, and 4 h, respectively. This same EO at 0.5 × MIC exhibited fungistatic activity against *C. glabrata* after 8 h. This EO, at 1 × MIC or 2 × MIC, displayed a fungicidal effect against *C. glabrata* after 12 h (Figure 1G).

Table 5 depicts the minimal biofilm-inhibiting concentration (MBIC) and the minimal biofilm-eradicating concentration (MBEC) obtained with the *Litsea cubeba*, *Citrus limon,* and *Cupressus sempervirens* EOs and gallic acid. The *Cupressus sempervirens* EO gave the best antibiofilm activity; the MBIC and MBEC values ranged between 62.5 and 1000 µg/mL against all the *Candida* species (Table 5). The lowest MBIC and MBEC values were achieved against *Candida krusei* at 62.5 and 250 µg/mL, respectively (Table 5).

The antifungal activity findings obtained in the in vitro assays were confirmed by using an in vivo infection model, namely the *Caenorhabditis elegans–Candida* infection assay, which is regarded as an infection model to study *Candida*-associated infections. Initially, the toxicity of the selected plant-derived products was evaluated by testing approximately 15–20 late-L4 larvae in each well of a 96-well microplate, exposed to concentrations of the selected products of 0.5 × MIC, 1 × MIC, and 2 × MIC at 25 °C for 24 h. The *Litsea cubeba* and *Cupressus sempervirens* EOs at concentrations between 31.25 and 125 μg/mL as well as gallic acid at concentrations between 15.62 and 250 μg/mL were not toxic (*p* > 0.05) against *C. elegans* as compared to untreated larvae (data not shown). In turn, the *Citrus limon* EO at 0.5 × MIC (125 μg/mL) was not toxic (*p* > 0.05), but this same EO at 1 × MIC (250 μg/mL) and 2 × MIC (500 μg/mL) was significantly toxic (*p* < 0.05 and *p* < 0.0001, respectively) (data not shown).

**Figure 2:**
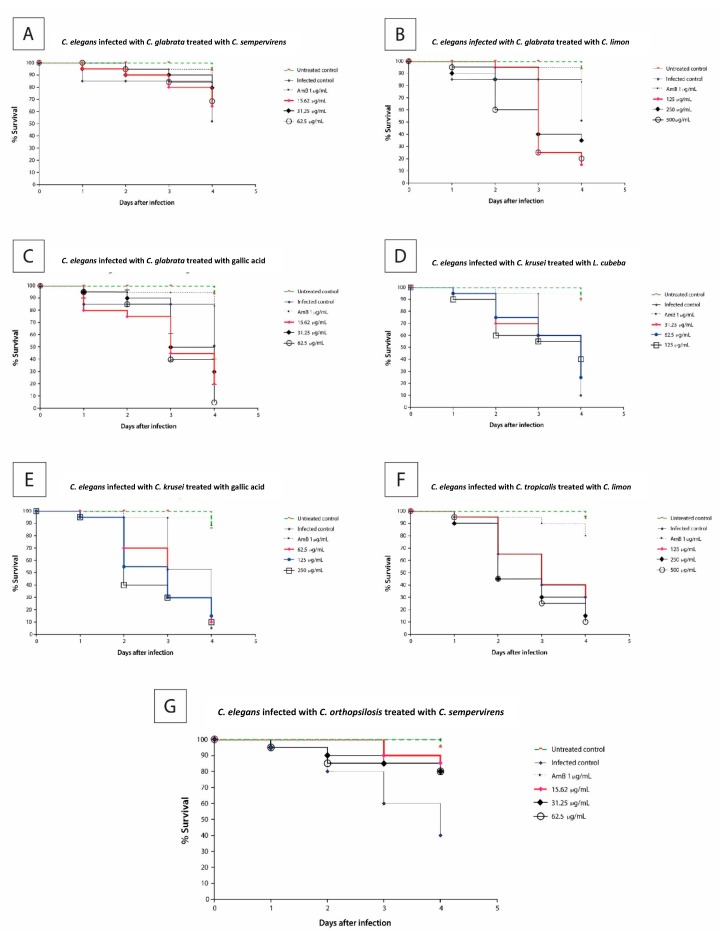
Survival curves of the responses to the tested compound concentrations from the *Caenorhabditis elegans–Candida* infected model. Nematode survival diminished at 0.5 × MIC, 1 × MIC, and 2 × MIC for all the compounds, except for the *Cupressus sempervirens* EO. (**A**)—*C. elegans* infected with *C. glabrata* and treated with *C. sempervirens* EO, (**B**)—*C. elegans* infected with *C. glabrata* and treated with *C. limon* EO, (**C**)—*C. elegans* infected with *C. glabrata* and treated with gallic acid, (**D**)—*C. elegans* infected with *C. krusei* and treated with *L. cubeba* EO, (**E**)—*C. elegans* infected with *C. krusei* and treated with gallic acid, (**F**)—*C. elegans* infected with *C. tropicalis* and treated with *C. limon,* (**G**)—*C. elegans* infected with *C. orthopsilosis* and treated with *C. sempervirens*. The untreated control group is represented by the green lines; the infected control, the fungicidal control drug (amphotericin B), and the different concentrations of the tested compounds are represented by symbols. The results were obtained from three independent experiments with at least three replicates.

The *C. glabrata*-infected larvae were treated with the *Cupressus sempervirens* (Figure 2A) and *Citrus limon* (Figure 2B) EOs and gallic acid (Figure 2C) at 25 °C for four days. Only in the presence of *Cupressus sempervirens* was a higher frequency of viable larvae maintained (Figure 2A). In contrast, the larvae infected with *C. krusei*, and treated with the *Litsea cubeba* EO (Figure 2D) at concentrations between 31.25 and 125 μg/mL or gallic acid (Figure 2E) at concentrations between 62.5 and 250 μg/mL, were not cured of candidiasis.

The *Citrus limon* EO at concentrations between 125 and 500 μg/mL was used to treat larvae infected with *C. tropicalis* (Figure 2F). After 24 h, a small percentage of dead larvae (5–10%) was detected. However, after 48 h, 35% and 55% of the larvae died at EO concentrations of 125 μg/mL and 250–500 μg/mL, respectively. At the end of four days, only 40% and 10–15% of the larvae survived, respectively, at the same concentrations.

Lastly, worms infected with *C. orthopsilosis* were treated with the *Cupressus sempervirens* EO (Figure 2G). Exposure to this EO (15.62 to 62.5 µg/mL) increased the survival of *C. elegans* worms infected with *C. orthopsilosis* as compared to the treated control. At four days postinfection, 80–85% of the infected and treated larvae survived.

## 3. Discussion

In Brazil, plant-derived products have gained importance because of the publication of Resolution 971 (3 May 2006) [13] and Act 5813 (22 June 2006) [14], which regulate the National Policy on Integrative and Complementary Practices and the National Policy on Medicinal and Phytotherapeutic Plants, respectively. These regulations introduced the use of medicinal plants and phytotherapeutic drugs into the Unified Health System (SUS) and aimed to ensure safe access of the Brazilian population to these medications as well as their rational application, promoting the sustainable use of the national biodiversity.

In this scenario, this study evaluated the antifungal potential of plant-derived products (essential oils, Brazilian native plant extracts, and plant constituents) that have been employed as antimicrobials in folk medicine. Among the EOs assessed herein, the *Cupressus sempervirens, Citrus limon,* and *Litsea cubeba* EOs are noteworthy. According to literature data, the EO extracted from *Cupressus sempervirens* leaves exhibits similar chemical composition to the composition identified here, albeit with different percentages. For instance, Selim et al. [15] reported that α-pinene (48.6%), δ-3-carene (22.1%), limonene (4.6%), and α-terpinolene (4.5%) are the main constituents of *Cupressus sempervirens* studied in Saudi Arabia, whereas Ibrahim et al. [16] described α-pinene (21.15%), terpinen-4-ol (6.98%), allo-ocimene (24.00%), and α-cedrol (23.68%) as the main components of Egyptian *Cupressus sempervirens*. As for *Cupressus sempervirens* growing in Brazil, we determined some of these substances at lower concentrations as well as the compounds sabinene (20.3%) and citral (20.0%), which were reported at higher concentrations in the EO extracted from *Cupressus sempervirens* leaves for the first time (Table 4).

The *Cupressus sempervirens* anticandidal activity against *C. albicans* has been demonstrated, but it has not been defined as fungistatic or fungicidal [17,18]. Here, we showed the greater susceptibility of *C. glabrata* and *C. orthopsilosis* to the *Cupressus sempervirens* EO (Table 1). These *Candida* species have been cited as causing a significant increase in *Candida* infections in the last few years [19]. Additionally, *C. glabrata* is the NAC species that has been the most commonly isolated from the environment and by health practitioners in a Brazilian Tertiary Hospital [20], and *C. orthopsilosis* has been identified as the prevalent organism among yeasts isolated from the hydraulic system of a hemodialysis facility [21].

Exposure to the *Cupressus sempervirens* EO completely inhibited *C. orthopsilosis* (Figure 1F) and *C. glabrata* (Figure 1G) cells. The fungicidal action of this EO could be partly attributed to its major constituents such as sabinene, citral, and terpinen-4-ol, which have been reported to display antimicrobial effects [22,23,24]. Moreover, at 2 x MIC, this EO provided the same effect as 4 μg/mL AMB against *C. orthopsilosis* (Figure 1F), which is the best antifungal drug concentration with fungicidal action that has been described in in vivo studies [25].

The *C. elegans* larvae infected with *C. glabrata* (Figure 2A) or *C. orthopsilosis* (Figure 2G) and treated with the *Cupressus sempervirens* EO at concentrations between 15.62 and 62.5 μg/mL had significantly (*p* < 0.05) higher survival as compared to the infected larvae control four days postinfection. This suggested that this EO might be a valuable antifungal agent against *Candida* infections. Besides that, this EO showed antibiofilm activity (MBIC or MBEC) against all the tested *Candida* non-*albicans* species (Table 5), which pointed out that it could be an adjuvant in the treatment of biofilm-associated *Candida*-non-*albicans* infections.

The *Citrus limon* EO exhibited anticandidal activity (MIC) against all the assayed strains (Table 1). At concentrations between 125 and 500 µg/mL and between 250 and 500 µg/mL, this EO displayed a fungicidal effect against *C. tropicalis* ATCC 13803 (Figure 1D) and *C. glabrata* ATCC 2001 (Figure 1E), respectively. The antifungal mechanism of the *Citrus limon* EO is associated with its main component, limonene [26], which was detected at a similar concentration to the concentration reported by Campelo et al. [27]. Limonene damages the *C. albicans* cell wall/membrane, thereby modifying cellular adhesion and plasticity, pH, and ionic content [26]. In addition, such damage causes oxidative stress and consequent DNA damage, resulting in cell cycle modulation and apoptosis as demonstrated by Thakre et al. [28]. The high concentration of limonene in the EO could contribute to its nematocidal effect [29].

The *Litsea cubeba* EO is known to possess diverse biological properties, among which the antimicrobial action is worthy of note [30]. Here, this EO afforded the best activity against *C. krusei* ATCC 6258, with MIC and MBIC values of 62.5 and 250 μg/mL, respectively. Its anti-*Candida* effect can be justified by the presence of chemical constituents such as limonene, citral, neral, terpinen-4-ol, and geraniol (Table 4), which have previously been described to present anti-*Candida* activity [26,31]. Moreover, in agreement with a previous study [32], we confirmed the *Litsea cubeba* EO nematocidal activity.

Gallic acid had great inhibitory action against *C. glabrata* and *C. krusei* (Table 1), but better antibiofilm activity against *C. krusei* (Table 5). Previous studies have shown antifungal activity for gallic acid against *C. albicans* and filamentous fungi [33,34,35,36]; however, its activity against *Candida* biofilms has been poorly investigated. This compound can inhibit biofilm formation [34], as confirmed by the *C. krusei* antibiofilm result (MBIC) recorded here. Gallic acid toxicity to *C. elegans* larvae has been reported at concentrations starting from 120 µg/mL [36].

The fungistatic and fungicidal actions of the EOs make them promising alternatives to treat superficial candidiasis; that is, to treat oral candidiasis and denture stomatitis by topical administration, since they can be included in mouth rinses or toothpastes [37]. Interestingly, we detected that all the assayed plant-derived products at 250 µg/mL had an effect on *C. krusei* biofilms (Table 5), suggesting that these products might be valuable antifungal agents in the therapy against *C. krusei* biofilm-associated infections. This organism is an important pathogenic *Candida* species that is frequently refractory to conventional antimicrobial agents and has been isolated from patients with oral candidiasis [38]. Further studies using an in vivo biofilm-associated animal model (e.g., a rat model of acute dermal toxicity) are necessary to confirm that the EOs might be useful to treat candidal biofilm-associated infections, especially topical infections.

## 4. Materials and Methods

### 4.1. Essential Oils

*Citrus reticulata* (peel), *Citrus reticulata Blanco* (peel and fresh and dry leaves), *Citrus reticulata* var. *cravo* (peel), *Cupressus sempervirens* (leaves), *Citrus limon* (L.) Burm (leaves), and *Litsea cubeba* (fruits) were harvested in Rio Verde (17°99.4′63.2′’ S and 51°05.2′44.6′’ W), GO, Brazil, on January 2nd, 2017, at 09:00. Voucher specimens (#CR-25, #CRB-25′, #CRC-25′’, #CS556, #CL89, and #LC2800) were deposited in the herbarium at the Instituto Federal Goiano (IF-GOIANO), in Rio Verde. Briefly, distilled water (500 mL) was added to the plant material (100 g) and transferred to a Clevenger-type apparatus. The essential oil (EO) was collected, and the remaining water was eliminated with anhydrous sodium sulfate, which was followed by filtration. This method was used in triplicate, and the obtained EOs were kept under refrigeration (4 °C). The mean quantities of the EOs (w/w) were obtained on the basis of the plant material weight and data from three experiments.

*Cananga odorata*, *Cedrus atlantica*, *Citrus aurantium* (Petitgrain), *Citrus sinensis* L. (peel), *Cymbopogon martinii*, *Cymbopogon nardus*, *Eucalyptus globulus*, *Eugenia caryophyllus*, *Melaleuca alternifolia*, *Mentha arvensis*, *Mentha piperita* L., *Origanum vulgare* L., *Pelargonium graveolens*, *Piper aduncum* L., and *Rosmarinus officinalis* EOs were purchased from FERQUIMA^®^ (Vargem Grande Paulista, SP, Brazil). *Betula pendula* Roth., *Citrus nobilis* (peel), and *Psidium cattleyanum* (fresh leaves) EOs were acquired from LASZLO^®^ (Belo Horizonte, MG, Brazil). *Cinnamomum zeylanicum* and *Cymbopogon citratus* (DC) Stapf EOs were obtained from AROMATERÁPICA^®^ (Sorocaba, SP, Brazil).

### 4.2. Plant Extracts

*Anacardium occidentale* L. and *Anacardium othonianum* cashew nuts were obtained from a local market in Franca (Oct/2013) and from Montes Claros de Goias (Mar/2017). Voucher specimens (SPFR 16040 and HJ3793) were deposited in the Biology Department Herbarium in the Plant Systematics Laboratory of the Faculty of Philosophy, Sciences and Letters of Ribeirão Preto, University of São Paulo, Brazil (Herbarium SPFR) and in the Herbarium Jataiense Germano Guarim Neto of the Goiás Federal University, Brazil (Herbarium HJ). The air-dried powdered *A. occidentale* and *A. othonianum* nuts were extracted with ethanol.

*Vochysia divergens* was collected in the Pantanal area, in the State of Mato Grosso (16°35′22″ S and 56°47′83″ W) in January 2017. A voucher specimen UFMT 39559 was deposited in the Herbarium of the Mato Grosso Federal University (UFMT), Brazil (Herbarium UFMT). The *V. divergens* stem barks were powdered and exhaustively extracted by maceration at room temperature; ethanol was employed. After filtration, the solvent was removed under reduced pressure to yield the ethanolic extract.

The *Curcuma longa* L. extracts were provided by Dr. Marco Túlio Menezes Carvalho, from the State University of Minas Gerais, MG, Brazil. The dried rhizomes were obtained from a local market (September 2017) in Passos, MG (20°43′13′’ S and 46°36′36′’ W), and the powdered material was stored in the dark. The extracts were obtained by maceration of the powdered curcuma rhizomes, at room temperature; boiling water or ethanol/water (50:50, v/v) was employed.

### 4.3. Plant-Derived Compounds

The plant-derived compounds gallic acid, caffeic acid, ferulic acid, benzoic acid, salicylic acid, menthol, and alpha-bisabolol were purchased from Sigma-Aldrich (St. Louis, MO, USA).

### 4.4. Candida Species

Reference strains of six *Candida* species, including *C. albicans* SC 5314, *C. glabrata* ATCC 2001, *C. parapsilosis* ATCC 22019, *C. krusei* ATCC 6258, *C. tropicalis* ATCC 13803, and *C. orthopsilosis* ATCC 96141 were used in this study. The strains were maintained at −70 °C in sterile distilled water plus 50% glycerol and subcultured in Sabouraud dextrose agar (SDA, Difco, Detroit, MI) and CHROMagar Candida medium (Becton Dickinson and Company, Sparks, MD) at 37 °C for 24 h to ensure purity and viability.

### 4.5. Minimum Inhibitory Concentration Determination

The in vitro antifungal susceptibility assays of all the natural products were performed by the broth microdilution method according to the adapted protocol M27-S4 from the Clinical and Laboratory Standards Institute [39]. Sterile microtiter plates (Corning Inc., NY, USA) were used. The inoculum size was 2.5 × 10^3^ cells/mL. The final concentration of amphotericin B (AMB) and the tested products ranged from 0.03 to 16 µg/mL and from 3.90 to 2.000 µg/mL, respectively. AMB and all the natural products were solubilized in DMSO (2%) and diluted in Roswell Park Memorial Institute (RPMI 1640, Sigma-Aldrich, St. Louis, MO, USA) medium added with 0.2% glucose. The *C. parapsilosis* ATCC 22019 and *C. krusei* ATCC 6258 strains and AMB were included as quality control [39]. The minimum inhibitory concentration (MIC) was determined with the fluorometric indicator resazurin at 0.01% (w/v) [40]. MIC was defined as the lowest antifungal/product concentration that maintained a blue hue. The AMB breakpoint adopted herein was ≤1 µg/mL, whereas AMB >1 µg/mL was considered as resistant [41]. The wells where micro-organism growth occurred displayed the pink color. Plant-derived products were considered active when MIC was <100 µg/mL, moderately active when MIC ranged from 100 up to 500 µg/mL, and weakly active when MIC was >500 and <1000 μg/mL. Above 1000 μg/mL, the products were considered inactive [42]. All the tests were conducted in triplicate.

### 4.6. Identification of the Chemical Composition of the EOs

Gas chromatography-flame ionization detection and gas chromatography–mass spectrometry analyses were accomplished with Shimadzu QP2010 Plus and GCMS2010 Plus (Shimadzu Corporation, Kyoto, Japan) systems. The GC-MS and GC-FID conditions and the identification of the chemical constituents of the EOs were carried out in agreement with the methodology proposed by Santos et al. [43].

### 4.7. Time-Kill Curves

All the compounds and natural products were evaluated at 0.5 × MIC, 1 × MIC, and 2 × MIC for each more susceptible *Candida* strain, at predetermined incubation time points (0, 2, 4, 6, 8, 12, 24, and 48 h). AMB was used at 4 µg/mL [25]. The *Candida* strains were subcultured on Sabouraud Dextrose Agar (SDA) plates at 35 °C for 24 h, suspended in 5 mL of RPMI 1640, and adjusted to a 0.5 McFarland turbidity standard (1 to 5 × 10^6^ cells/mL) with a nephelometer (ATB 1550, BioMérieux, France). Next, the micro-organism suspension was diluted in RPMI 1640, to give a new suspension containing 2.5–5.0 × 10^4^ cells/mL, and the plant product was added at an appropriate concentration. The latter suspension was incubated at 35 °C for 48 h, and 100 μL aliquots were removed at each time point. Tenfold serial dilutions were performed, and 10 µL aliquots were plated on SDA plates and incubated at 35 °C for 24 h. The mean colony-forming unit (CFU) value was converted to the respective log CFU/mL value. The data were plotted as log CFU/mL against time point. A fungicidal activity was considered to exist when micro-organism growth decreased by at least 3 log_10_ CFU/mL as compared to the initial inoculum, to result in a reduction of 99.99% of CFU/mL. In turn, a fungistatic activity was considered to exist when micro-organism growth was less than 99.9% or <3 log_10_ in CFU/mL as compared to the initial inoculum [22,44]. The experiments were conducted in triplicate.

### 4.8. Evaluation of the Effects on Biofilm

Two groups of tests were completed to evaluate the activity of the tested plant products against *Candida* biofilms: i) inhibition of biofilm formation was assessed to determine the minimal biofilm-inhibiting concentration (MBIC) and ii) effect on the preformed biofilm was analyzed to determine the minimal biofilm-eradicating concentration (MBEC). Three EOs, namely the *Litsea cubeba*, *Citrus limon*, and *Cupressus sempervirens* EOs, and one plant-derived compound (gallic acid) were selected after MIC determination. A sterile 96-well flat-bottom microtiter plate (Corning) was used. Inhibition/eradication of biofilm formation by the *Candida* strains was assayed according to a previously described methodology [45]. The EOs and gallic acid (concentration range from 1.95 to 2000 μg/mL) were dissolved in DMSO and two-fold diluted in RPMI 1640 medium at 35 °C for 48 h. Biofilm viability was measured by the tetrazolium salt (sodium 3′- [1-(phenylaminocarbonyl)-3,4-tetrazolium]-bis(4-methoxy-6-nitro) hydrated benzene sulfonic acid, XTT) reduction assay, by adding 100 μL of XTT-menadione to each well. Wells containing culture medium/biofilms/XTT/menadione (positive control) and wells containing culture medium/XTT/menadione (negative control) were included. The optical density (OD) was read on a microtiter plate reader (Asys - Eugendorf, Salzburg, Austria) at a wavelength of 492 nm [45]. The effective concentration of the EO/chemical compound capable of reducing ≥90% OD as compared to the control free of a chemical substance (100% of survivors) was considered as the MBIC or MBEC. Each experimental condition was tested in triplicate, and the arithmetic mean of the results was used to present the results.

### 4.9. In Vivo Toxicity to Caenorhabditis elegans

Acute toxicity was measured after exposure of *Caenorhabditis elegans* to the tested plant product for 24 h. The AU37 [glp-4(bn2) I; sek-1(km4) X] mutant strain was kindly provided by the São Paulo State University, Dr. Júlio de Mesquita Filho, Instituto de Ciência e Tecnologia, Departamento de Biociências e Diagnóstico Bucal, São José dos Campos, SP, Brazil. Nematodes grew on nematode growth medium (NGM) agar plates, seeded with *Escherichia coli* OP50 and incubated at 16 °C. They were synchronized by treatment with sodium hypochlorite, transferred, and incubated in NGM without *E. coli* OP50. The worms were then washed with NaCl 50 mM. Around 20 worms were added to the wells of 96-well microplates containing culture broth (60% 50mM NaCl, 40% BHI (brain heart infusion broth), cholesterol (10 μg/mL), kanamycin (90 µg/mL), and ampicillin (200 µg/mL)). Selected plant-derived products were added to each well at 0.5 × MIC, 1 × MIC, or 2 × MIC; AMB was added at 1.0 µg/mL. The 96-well microplates were maintained at 25 °C, and individual worm survival was assessed after 24 h, Nematodes were considered dead when they were rod-shaped and did not respond to touching [46,47]. Two independent experiments were carried out for each treatment.

### 4.10. Infection Assay of Caenorhabditis elegans–Candida Species

*C. elegans* AU37 fed with *E. coli* OP50 were maintained at 16 °C and synchronized as described above. *Candida* species were grown on BHI-agar, and worms in stage L4 were added to the plates containing each *Candida* species. Next, the plates with *Candida* and worms were incubated at 25 °C for 2 h. Then, the worms were washed with 50 mM NaCl, and *C. elegans* suspension was adjusted to contain 15–20 L4 larvae per well in a 96-well microplate [46]. In each well, the plant product was added at 0.5 × MIC, 1 × MIC, or 2 × MIC concentrations. The plates were incubated at 25 °C for four days. Worms not treated with a plant-derived product and worms infected with *Candida* species served as controls. Worm survival was expressed as a percentage of worm viability at day zero. Three independent experiments with at least three replicates were performed.

## 5. Statistical Analysis

Statistical analyses were done with the Program GraphPad Prism 5.0 (GraphPad Software Inc., San Diego, CA, USA). The survival curve of *C. elegans* was plotted by using the Kaplan–Meier method, and the survival differences were analyzed by log-rank (Mantel–Cox). A value of *p* < 0.05 was considered statistically significant.

## Figures and Tables

**Table 1:** Minimal Inhibitory Concentration (µg/mL) values of essential oils obtained against *Candida* species.

Essential Oils	*Candida albicans* SC 5314	*Candida tropicalis* ATCC 13803	*Candida krusei* ATCC 6258	*Candida glabrata* ATCC 2001	*Candida parapsilosis* ATCC 22019	*Candida orthopsilosis* ATCC 96141
*Betula pendula* Roth.	>2000	>2000	>2000	>2000	>2000	>2000
*Cananga odorata*	>2000	>2000	>2000	>2000	2000	>2000
*Cedrus atlantica*	>2000	>2000	>2000	>2000	>2000	>2000
*Cinnamomum zeylanicum* (leaves)	500	500	500	500	500	500
*Citrus aurantium* (Petitgrain)	>2000	>2000	>2000	>2000	>2000	>2000
*Citrus limon* (L.) Burm	500	250	500	250	500	500
*Citrus nobilis* (peels)	2000	>2000	>2000	2000	>2000	>2000
*Citrus reticulata* (peels)	2000	1000	250	1000	1000	250
*Citrus reticulata* Blanco (dry leaves)	>2000	>2000	>2000	2000	2000	2000
*Citrus reticulata Blanco* (peels)	1000	2000	500	1000	1000	1000
*Citrus reticulata* var. *cravo* (peels)	>2000	>2000	>2000	2000	>2000	2000
*Citrus reticulata* Blanco (fresh leaves)	2000	2000	>2000	>2000	>2000	>2000
*Citrus sinensis L* (peels)	>2000	>2000	>2000	>2000	>2000	>2000
*Cupressus sempervirens* (leaves)	250	250	62.5	31.25	62.5	31.25
*Cymbopogon citratus* (DC) Stapf	2000	>2000	2000	2000	>2000	>2000
*Cymbopogon martinii*	2000	2000	2000	2000	2000	2000
*Cymbopogon nardus*	2000	2000	2000	2000	2000	2000
*Eucalyptus globulus*	>2000	>2000	1000	2000	>2000	>2000
*Eugenia caryophyllus*	>2000	>2000	2000	2000	2000	2000
*Litsea cubeba*	500	1000	62.5	250	500	250
*Melaleuca alternifolia*	>2000	>2000	2000	>2000	>2000	>2000
*Mentha arvensis* L.	2000	2000	2000	2000	2000	2000
*Mentha piperita* L.	>2000	>2000	>2000	>2000	>2000	>2000
*Origanum vulgare* L.	500	500	1000	500	1000	1000
*Pelargonium graveolens*	>2000	>2000	2000	2000	2000	>2000
*Piper aduncun* L. (leaves)	>2000	>2000	>2000	>2000	>2000	>2000
*Piper aduncun* L. (inflorescences)	>2000	>2000	>2000	2000	>2000	2000
*Piper aduncun* L. (branches)	2000	>2000	>2000	2000	2000	2000
*Psidium cattleyanum* (dry leaves)	>2000	>2000	>2000	>2000	>2000	>2000
*Rosmarinus officinalis*	>2000	>2000	>2000	>2000	>2000	>2000

**Table 2:** Minimal inhibitory concentrations (MIC) of plant extracts against *Candida* species.

Plant Extracts	*Candida* Species (MIC µg/mL)					
	*C. albicans* ATCC 5314	*C. tropicalis* ATCC 13803	*C. krusei* ATCC 6258	*C. glabrata* ATCC 2001	*C. parapsilosis* ATCC 22019	*C. orthopsilosis* ATCC 96141
*Anacardium occidentale* (ethanolic extract)	>2000	>2000	>2000	>2000	>2000	>2000
*Anacardium othonianum* (ethanolic extract)	>2000	>2000	>2000	>2000	>2000	>2000
*Curcuma longa* (ethanolic extract)	>2000	>2000	>2000	>2000	>2000	>2000
*Curcuma longa* L. (aqueous extract)	>2000	>2000	>2000	>2000	>2000	>2000
*Vochysia divergens* stem (ethanolic extract)	>2000	-*	>2000	>2000	>2000	-*

-*: They were not evaluated.

**Table 3:** Minimal inhibitory concentration (MIC) of plant-derived compounds against *Candida* species.

Compounds	*Candida* Species/MIC (µg/mL)					
	*C. albicans* SC5314	*C. tropicalis* ATCC 13803	*C. krusei* ATCC 6258	*C. glabrata* ATCC 2001	*C. parapsilosis* ATCC 22019	*C. orthopsilosis* ATCC 96141
Alpha-bisabolol	>2000	>2000	2000	>2000	>2000	>2000
Benzoic acid	>2000	>2000	>2000	>2000	>2000	>2000
Caffeic acid	>2000	>2000	1000	500	>2000	500
Ferulic acid	>2000	>2000	>2000	>2000	>2000	>2000
Gallic acid	500	1000	125	31.25	250	250
Menthol	>2000	>2000	>2000	>2000	>2000	>2000
Salicylic acid	>2000	>2000	>2000	>2000	>2000	>2000

**Table 4:** Chemical composition of essential oils (EOs) from *Cupressus sempervirens* and *Citrus limon* leaves and *Litsea cubeba* fruits.

Compounds	% RA			
	RI	*C. sempervirens*	*C. limon*	*L. cubeba*
*α*-Pinene	934	8.0	-	2.0
Sabinene	969	20.3	-	1.3
*β*-Pinene	974	-	-	2.0
Myrcene	991	6.0	-	1.3
δ-2-Carene	1001	4.0	-	-
*p*-Cymene	1023	5.0	-	-
Limonene	1024	3.9	53.4	37.0
*cis*-Limonene oxide	1129	-	2.0	-
*trans*-Limonene oxide	1133	-	7.0	-
*γ*-Terpinene	1054	4.0	-	-
Citronelol	1150	-	3.8	-
Linalool	1095	-	1.9	4.0
Terpinen-4-ol	1177	15.4	-	-
α-Terpineol	1186	2.4	-	2.3
Neral	1238	5.0	11.0	31.4
Citral	1249	20.0	-	12.0
Geraniol	1268	-	9.0	1.2
Nerol	1363	-	6.0	-
Geraniol acetate	1384	-	4.0	-
*β*-Caryophyllene	1415	-	-	1.7
*δ*-Cadinene	1522	3.0	-	-
Cedrol	1598	2.1	-	-
	**Total**	**99.1**	**98.1**	**96.2**

RI: Retention index; % RA: relative area percentage.

**Table 5:** Activity of the essential oils and gallic acid on biofilm formation and against preformed *Candida* species biofilms.

Compounds	*Candida* Species (µg/mL)											
	*C. albicans* SC 5314		*C. glabrata* ATCC 2001		*C. tropicalis* ATCC 13803		*C. krusei* ATCC 6258		*C. parapsilosis* ATCC 22019		*C. orthopsilosis* ATCC 96141	
	MBIC *	MBEC **	MBIC *	MBEC **	MBIC *	MBEC **	MBIC *	MBEC **	MBIC *	MBEC **	MBIC *	MBEC **
*Citrus limon* (L.) Burm	2000	1000	1000	1000	2000	1000	125	250	2000	1000	1000	1000
*Cupressus sempervirens*	1000	1000	250	1000	500	1000	62.5	250	500	1000	125	1000
Gallic acid	>2000	>2000	>2000	>2000	>2000	>2000	250	500	500	>2000	>2000	>2000
*Litsea cubeba*	2000	1000	2000	1000	2000	1000	250	1000	1000	1000	2000	1000

* MBIC: minimum biofilm formation inhibiting concentration (capable of reducing ≥90% optical density (OD) compared to the control free of chemical substances); ** MBEC: minimum biofilm-eradicating concentration (capable of reducing ≥ 90% OD compared to the control free of chemical substances).
